# Analyzing EFL learners’ demotivating factors in blended learning context

**DOI:** 10.3389/fpsyg.2023.1290034

**Published:** 2023-10-20

**Authors:** Wenjing Zhou, Yan Zhan, Esther Jawing

**Affiliations:** ^1^School of Foreign Languages, Jiangxi Agricultural University, Nanchang, China; ^2^Centre for the Promotion of Knowledge and Language Learning, Universiti Malaysia Sabah, Kota Kinabalu, Malaysia

**Keywords:** EFL learner, demotivating factors, blended learning context, group differences, college students

## Abstract

In second language and foreign language learning, demotivation in learning is a common occurrence. Almost all previous studies on learners’ demotivation focused on traditional classroom learning environment rather than blended learning setting. This paper investigates learner perceptions of demotivating factors for Chinese EFL college students in blended learning context. 272 college sophomores with varied majors from a university in Mid-East China took part in a questionnaire survey. The questionnaire, consisted of 34 4-point Likert type items about learners’ demotivation in a blended EFL learning environment, was adapted from Kikuchi’s demotivation questionnaire and Xie’s LPDS (Learner Perceptions of Demotivator Scale). An exploratory factor analysis was performed to explore the factor structure of the questionnaire items. Then mean scores of items loading on each factor were calculated and independent samples t-test analysis was adopted to examine the differences of demotivating factors between different groups of participants. Five demotivating factors from the questionnaire were extracted. The findings reveal a newly discovered factor: learners’ lack of self-discipline in online learning. The paper indicates that there is no significant difference of these five demotivating factors between male and female learners, and between rural and urban learners. Whereas less motivated learners perceive four among the five factors to be more demotivating than more motivated learners.

## Introduction

Learner’s motivation has always been a popular topic in the second language acquisition research, whereas much less has been investigated in the other side, the demotivation aspect, which is equally important and worth-caring. Zoltán Dörnyei is one of the earliest researchers that focused on learner demotivation study, and he concluded demotivation as “specific external forces that reduce or diminish the motivational basis of a behavioral intention or an ongoing action” (Dörnyei and Ushioda, 2011). Many other researchers (Falout et al., 2009; Sakai and Kikuchi, 2009; Zhou and Wang, 2012; Kikuchi, 2015; Xie et al., 2021) believed demotivation included both external factors (such as negative teacher behaviors and inappropriate teaching materials) and internal factors (such as low expectancy for success and lacking self-confidence). In this study, demotivation is considered to contain both external and internal factors which reduce or diminish the motivation to study English, a widely accepted definition.

Research on demotivating factors in classes can be traced back to 1990s in the United States (Gorham and Christophel, 1992; Christophel and Gorham, 1995; Gorham and Millette, 1997) in instructional communication. Gorham and Christophel (1992) identified 20 categories of demotivating factors among 2,404 motivating and demotivating factors listed by 308 college students at an American university. These factors mainly concerned context, structure/format, and teacher behavior. The results indicated that negative teacher behaviors were considered crucial to students’ demotivation, thus motivation decrease was perceived as a teacher-owned problem. Later, Christophel and Gorham (1995) investigated the changes in 319 students’ perceptions of demotivating factors after one semester in college classes at American universities; Gorham and Millette (1997) compared responses from 224 teachers at an American university and responses from 308 undergraduate students who were surveyed in a previous study (Gorham and Christophel, 1992) to investigate their perceptions of motivating and demotivating factors in college classrooms, and both studies echoed the previous finding by Gorham and Christophel (1992): the students tended to attribute demotivation in classes as a teacher-owned problem. In another research by Zhang (2007), colleges students from four countries: the United States, China, Germany, and Japan were surveyed to investigate learning demotivating factors in classes. The results indicated that teachers’ incompetence was perceived as the most demotivating factor in their English language learning across four countries. This finding was also echoed by (Katt and Condly, 2009) in their study on classroom motivating and demotivating factors.

In later years, more research was implemented on learner perceptions of demotivating factors under learning English as a Foreign Language (EFL) context, especially in Eastern Asia culture. To investigate the sources of demotivation among Japanese EFL learners, Sakai and Kikuchi (2009) conducted a quantitative study among 656 Japanese high school learners of English through a 35-item questionnaire developed by themselves based on reviewing previous studies. They extracted five demotivators (demotivating factors): (1) learning contents and materials, (2) teachers’ teaching styles, (3) inadequate school facilities, (4) lack of intrinsic motivation, (5) test scores. The results indicated that the learning contents & materials and test scores factors ranked the highest demotivating factors for many Japanese high school students, whereas neither teacher competence nor teaching styles was a strong cause of demotivation. In another survey by Kikuchi and Sakai (2009) with 112 EFL university students who were asked to recall their high learning experience, 5 different factors were extracted including (1) course books, (2) inadequate school facilities, (3) test scores, (4) non-communicative methods, (5) teachers’ competence and teaching styles. They found that course books and non-communicative methods were the most demotivating factors, whereas inadequate school facilities as a less demotivating factor. Kikuchi (2011) further developed his 35-item questionnaire into a six-factor demotivation model (36 items) to investigate demotivation on 1,334 Japanese high school EFL students. The demotivating factors consisted of teacher behavior, characteristics of classes, class environment, class materials, experience of failure and loss of interest. The findings indicated that pedagogical materials and class environment were generally stronger demotivators than teacher behavior. He also found that less motivated learners considered class environment and loss of interest as more demotivating than more motivated learners, and the male learners were more sensitive to the learning environment as demotivators.

Kikuchi’s work on EFL learners’ demotivation inspired many other researchers. His six-factor demotivation model was adopted by Krishnan and Pathan (2013) to investigate the demotivating factors of Pakistani EFL undergraduates, and their findings supported (Sakai and Kikuchi, 2009) framework and suggested a new factor - negative attitude of society toward English language - as demotivator. Çankaya (2018) adopted this demotivation model to explore the main factors causing demotivation in EFL learning among university students of vocational school, and the findings indicated that inadequate class materials were not perceived as demotivating and teacher competence was not a very strong cause of demotivation. Xie et al. (2018) utilized Kikuchi’s demotivation questionnaire (Kikuchi, 2015) to measure learner perceptions of demotivation among college EFL learners in China, and the results suggested that these demotivating factors were negatively correlated with learners’ performance, and male students were more demotivated than females over all the factors. Adara et al. (2021) employed a questionnaire adapted from Sakai and Kikuchi (2009) to investigate the differences of demotivating factors between public and private high school EFL learners during the Covid-19 pandemic, and their findings indicated that both groups of learners are mostly demotivated by inadequate school facilities, test scores, and teachers’ competence and teaching styles. Huwari et al. (2023) used a questionnaire adapted from Sakai and Kikuchi (2009) to investigate the elements that influence Jordanian undergraduate students’ acquisition of English as a Foreign Language (EFL), and the results showed that classroom environment was the most demotivating factors impacting Jordanian EFL undergraduate learners, whereas lack of self-confidence and interest were the least demotivating factors.

From the above studies, it can be discovered that research on learner perceptions of demotivation initiated in American college classrooms under L1 (first language) setting, and early research tended to attribute learners’ demotivation in classes to external factors (e.g., teacher behaviors). Then the research developed and flourished into learning English as a Second Language (EFL) classrooms in various types of schools (e.g., high schools, vocational schools, universities) in other countries. The findings of later research indicated that learners’ demotivation can be caused by both external factors (e.g., learning materials, class environment, test scores) and internal factors (e.g., loss of interest), whereas teacher behaviors were not perceived as strong demotivating factor by participants in many researches. Differences on demotivating factors among groups of learners (e.g., male and female learners, more motivated and less motivated learners, learners from different countries or types of schools) were also compared and analyzed in many later studies. It can be found that previous studies well shed light on learners’ perceptions of demotivating factors in L1 or learning EFL context, as well as differences on demotivating factors among various groups of learners.

However, to the best knowledge of the authors, almost all the previous studies are confined to traditional learning classrooms, whereas learners’ learning environments have been under reconstruction with the progress of world economy and society. Modern technology has been involved more and more in education field, especially the internet has been utilized commonly in classroom teaching and learning worldwide. One typical trend is that blended learning, which combines face-to-face learning and online learning, has been developed and implemented by various kind of schools throughout the world (Watson, 2008; Mozelius, 2017). The COVID-19 pandemic factually accelerated and strengthened this trend. Learning through blended way has become vital during the COVID-19 pandemic (Adedoyin and Soykan, 2023). In United Kingdom, a large majority (88.5%) of universities adopted a ‘blended-learning’ approach in their teaching over the 2020/21 academic year (Finlay et al., 2022). In China, all the Chinese universities and colleges adopted online learning or blended learning to fulfill education course during the pandemic (Zhao et al., 2022). This blended learning trend is likely to be maintained and applied globally in the post-pandemic era (Qadriani, 2022), and it is proposed by many educators as the most suitable teaching approach in the coming new era (Yang et al., 2022). Considering the popularity of blended learning worldwide, in order to help both teachers and students achieve better education outcomes, it is necessary to understand how to motivate learners and what demotivate them to study in blended learning environment, which has rarely been referred to till now. Thus, to help fill the gap, we propose the following research questions:

1. What are the demotivating factors for Chinese EFL college students in blended learning environment?
2. Do the demotivating factors differ for English learning motivation (less motivated and more motivated), gender (male and female), and family background (rural and urban)?

## Materials and methods

### Participants

272 college sophomores with varied majors from a university in Mid-East China took part in the research. Among them, about 41% (*n* = 111) were males and 59% (*n* = 161) were females. The participants were selected by cluster sampling, and they have been studying English under a blended learning context for one and a half years in the university. According to the data from the university website, this university consists of 17 schools with disciplines covering nine major categories and it has 23,676 full-time students, among which about 45% are males and 55% are females in September 2022. In the university, like most universities in China, a foreign language course is a compulsory one for freshmen and sophomores. About 95% students choose English for it, and 5% students select other languages. For those 95% students, they are expected to refer to online tools (several online Apps) to conduct preparing (self-learning), doing assignments and course discussions, specifically about 60% of the learning time is conducted online. According to Allen et al. (2007), a course can be considered in the form of blended when the portion of e-learning is at 30–79% range. Thus, they have been conducted EFL learning under a blended context.

### Instrument

The questionnaire (see Appendix A), adapted from Kikuchi’s demotivation questionnaire (Kikuchi, 2015) and LPDS (Learner Perceptions of Demotivator Scale; Xie et al., 2021), consisted of 34 4-point Likert type items about learners’ demotivation in a blended EFL learning environment. These items were designed to measure four factors: negative teacher behavior (7 items), loss of task value (9 items), low expectancy for success (8 items), and problems with learning environment (10 items). Participants are expected to choose one number from 1 to 4 (1 = Strongly Disagree / Not Demotivating at all; 2 = Disagree / Not Demotivating; 3 = Agree / Demotivating; 4 = Strongly Agree / Very Demotivating), and a higher score indicates a higher level of demotivation.

The questionnaire also included a question about learners’ motivation to learn English: How motivated are you to learn English? Four options are provided: (1). I have almost no motivation; (2). I have a little motivation; (3). I have moderate motivation; and (4). I have high motivation. Based on the replies to this question, the participants were divided into less motivated learners (participants who chose 1 or 2) and more motivated learners (participants who chose 3 or 4). Questions eliciting the participants’ gender (male or female), family background (rural or urban), etc. were also included in the questionnaire.

### Process

The questionnaire survey was done during the normal academic semester in June 2022 in a comprehensive university in Jiangxi province, PRC. The research questionnaires were administered to the participants during a 30-min class break to ensure that they have sufficient time. A teacher was there to instruct the participants to complete the questionnaires. Before the survey, a teacher thanked all the students of each class for their participation and assured them that their responses would be confidential and anonymous. The participants turned in the questionnaire paper when they finished them, and they can choose to withdraw anytime if they like. Finally, 272 questionnaires were received, 16 of which being considered disqualified due to too much missing information, so all together data of 256 samples was collected. Data from the questionnaires was filled into an Excel form, then input into SPSS 23 to be analyzed.

### Statistical analyses

Firstly, the data were analyzed using descriptive statistics to show the profile of the questionnaire items. Then, an exploratory factor analysis was performed to explore the factor structure of the questionnaire items. Following this, mean scores of items loading on each factor were calculated and independent samples t-test analysis was adopted to examine whether there are differences between less motivated and more motivated learners, between learners of different genders (male and female) and between learners from different family backgrounds (urban and rural).

## Results

### Demotivating factors for blended learning among EFL Chinese learners

Table 1 shows the descriptive statistics for each item. It can be seen that most means of the items are between 2.00 and 3.00, except for items 10 to 15 (all concerning learner’s task value) lower than 2.00 and item 27 (“I seldom have opportunities to practice English.”) barely above 3.00. More than half of the participants choose 3 (agree) or 4 (strongly agree) for the following items: items 18, 20 to 25 (concerning learner’s expectancy for success), items 26 to 30, 32 to 34 (concerning learner’s learning environment), items 36 to 40 (concerning teacher behavior), which means these items are perceived as more demotivating by participants.

**Table 1:** Descriptive statistics for participants’ questionnaire responses (N = 256).

No	*Max*	*Min*	*M*	*SD*	*Skewness*	*Kurtosis*	1(%)	2(%)	3(%)	4(%)
8	4	1	2.41	0.78	−0.11	−0.46	12.1	40.2	41.8	5.9
9	4	1	2.28	0.93	0.25	−0.78	21.9	39.5	27.7	10.9
10	4	1	1.77	0.68	0.54	0.11	35.9	52.0	10.9	1.2
11	4	1	1.86	0.71	0.41	−0.28	32.0	51.2	15.6	1.2
12	4	1	1.62	0.65	0.82	0.82	45.7	47.7	5.5	1.2
13	4	1	1.65	0.65	0.67	0.22	43.8	48.0	7.4	0.8
14	4	1	1.84	0.71	0.63	0.53	31.6	54.7	11.3	2.3
15	4	1	1.89	0.74	0.41	−0.34	31.3	49.6	17.6	1.6
16	4	1	2.02	0.81	0.32	−0.61	28.5	44.1	24.2	3.1
17	4	1	2.30	0.88	0.18	−0.66	18.8	41.4	30.9	9.0
18	4	1	2.61	0.76	−0.15	−0.30	7.0	35.5	47.3	10.2
19	4	1	2.38	0.77	−0.03	−0.42	12.1	43.4	39.1	5.5
20	4	1	2.59	0.83	−0.11	−0.51	9.4	35.2	43.0	12.5
21	4	1	2.61	0.81	−0.22	−0.39	9.0	32.4	46.9	11.7
22	4	1	2.70	0.82	−0.28	−0.35	7.8	28.9	48.4	14.8
23	4	1	2.77	0.80	−0.57	0.06	8.6	20.3	56.3	14.8
24	4	1	2.68	0.75	−0.30	−0.10	6.3	30.5	52.7	10.5
25	4	1	2.79	0.75	−0.23	−0.23	4.3	28.5	51.6	15.6
26	4	1	2.86	0.81	−0.50	−0.03	6.6	20.7	53.1	19.5
27	4	1	3.06	0.68	−0.53	0.65	2.3	13.3	60.2	24.2
28	4	1	2.80	0.81	−0.20	−0.49	5.1	29.3	46.1	19.5
29	4	1	2.66	0.80	−0.10	−0.44	6.6	34.8	44.9	13.7
30	4	1	2.66	0.77	−0.21	−0.27	6.6	32.4	49.2	11.7
31	4	1	2.43	0.81	0.00	−0.51	12.5	40.6	38.7	8.2
32	4	1	2.54	0.76	−0.28	−0.29	9.4	34.8	48.8	7.0
33	4	1	2.55	0.82	0.06	−0.53	8.6	40.6	38.3	12.5
34	4	1	2.67	0.81	−0.20	−0.42	7.8	31.6	46.5	14.1
35	4	1	2.39	0.80	−0.11	−0.55	14.1	39.1	41.0	5.9
36	4	1	2.71	0.78	−0.29	−0.22	6.6	29.3	50.8	13.3
37	4	1	2.79	0.83	−0.51	−0.13	8.6	21.1	52.7	17.6
38	4	1	2.86	0.89	−0.53	−0.35	9.4	19.1	47.3	24.2
39	4	1	2.51	0.85	0.02	−0.61	11.3	38.7	37.5	12.5
40	4	1	2.57	0.85	−0.11	−0.57	10.5	34.8	41.8	12.9
41	4	1	2.44	0.85	0.05	−0.61	13.3	39.8	36.3	10.5

Note: The standard error of skewness is 0.15; the standard error of kurtosis is 0.30.

A factor analysis using main component method was performed on the 34 items of the questionnaire. The results show that Kaiser-Meyer-Olkin (KMO) Measure of Sampling Adequacy is 0.864, and Bartlett’s Test of Sphericity (Chi-Square = 3881.090, *df* = 406, *p* < 0.001) is significant (see Table 2), which indicate that the questionnaire fits for factor analysis. Based on the scree plot (see Appendix B) and the interpretability of the factor solution, a five-factor (29 items) solution was extracted. After rotation, the five-factor structure accounts for 61.83% of the variance in total scores, and the communalities of all items are above 0.40. Similarly, factors loadings for all the items within each factor are above 0.40. Table 3 shows the pattern structure of the analysis and the factor loading on each item. The first three factors well meet the pre-assumed four-factor structure. Factor one contains eight items (items 18 to 25) concerning learners’ low expectancy for success. Factor two also contains eight items (items 9 to 16) concerning learners’ loss of task value. Factor three contains six items (items 36 to 41) concerning negative teacher behaviors. In the pre-assumed structure, factor four consists of 10 items concerning learning environment. However, 3 items (item 30: Sometimes, I played games or browse webpages online in studying time; item 31: Sometimes, I skipped online teaching and learning unintentionally; item 32: Sometimes, I played games or browse webpages online in studying time.) are separated from the factor through the analysis. It can be discovered that these items focus on learners’ self-discipline, so they are named “lack of self-discipline in learning online” as a new factor in this study. Thus, four items (items 26 to 29) are still retained in the actual fourth factor focusing on inappropriate learning environment, and the fifth factor is made of 3 items (items 30 to 32) relating to learners’ lack of self-discipline in learning online.

**Table 2:** KMO and Bartlett’s test.

KMO		0.864
Bartlett’s test of sphericity	Chi-square	3881.090
	*df*	406
	*p*	0.000

**Table 3:** Factor analysis of demotivation questionnaire (34 items).

No	Items	F1	F2	F3	F4	F5	communalities
Factor 1: Low Expectancy for Success							
21	I really want to master English, but I do not know how.	**0.873**	0.119	−0.02	0.098	0.055	0.789
22	I have not found an effective way to learn English.	**0.83**	0.15	0.049	0.081	0.138	0.74
19	I have made many attempts to learn English, but I have not improved.	**0.72**	0.175	−0.059	0.145	−0.066	0.579
20	I’m not aware of the strategies to improve my listening skills.	**0.709**	0.123	0.003	0.211	0.097	0.573
23	English grammar is tough and confusing.	**0.705**	0.086	0.141	0.058	0.254	0.591
24	Reading comprehension articles are hard to understand.	**0.682**	0.201	0.179	0.015	0.233	0.592
18	I seriously do not know how to speak English fluently.	**0.572**	0.297	−0.032	0.276	−0.037	0.495
25	I struggle with improving my English writing skills.	**0.554**	0.114	0.058	0.337	0.014	0.437
Factor 2: Loss of Task Value							
13	English has no use for my major.	0.051	**0.765**	0.087	0.156	−0.109	0.631
15	It’s not clear to me why I must learn English.	0.117	**0.758**	0.062	0.102	0.167	0.63
12	I wonder why English is needed in a monolingual country.	0.1	**0.729**	−0.016	0.12	−0.079	0.563
11	Learning English takes forever, and it may not get you anywhere.	0.137	**0.718**	−0.045	0.092	−0.018	0.546
10	I do not see the value of learning English.	0.1	**0.713**	−0.06	−0.001	0.238	0.579
14	I’m not interested in English at all.	0.276	**0.707**	−0.015	0.022	0.178	0.609
16	I take English class only because it’s a required class.	0.222	**0.701**	−0.035	0.108	0.131	0.571
9	The only purpose of learning English is to pass all the exams.	0.19	**0.508**	0.004	−0.014	0.378	0.437
Factor 3: Negative Teacher Behavior							
38	Teachers do not have faith in their students.	0.049	0.015	**0.815**	−0.124	0.135	0.7
37	Teachers do not have a sense of responsibility for the teaching job.	0.04	−0.048	**0.806**	−0.021	0.198	0.694
39	Teachers are not inspiring or encouraging.	0.067	0.036	**0.804**	−0.036	−0.089	0.662
41	Teachers seldom motivate us to learn.	−0.042	0.022	**0.796**	0.03	−0.039	0.638
36	Teachers are not responsive to our learning needs.	0.007	−0.047	**0.773**	0.053	0.158	0.628
40	Teachers reward performance rather than learning.	0.092	−0.007	**0.759**	0.035	−0.115	0.599
Factor 4: Inappropriate Learning Environment							
27	I seldom have opportunities to practice English.	0.209	0.065	−0.06	**0.814**	0.076	0.719
28	I do not collaborate with classmates in learning English.	0.116	0.048	0.023	**0.787**	0.175	0.666
29	I do not communicate with classmates about English learning.	0.189	0.191	−0.02	**0.706**	0.26	0.638
26	I do not deal with real language situations under blended teaching and learning environment.	0.266	0.185	−0.012	**0.703**	0.155	0.624
Factor 5: Lack of Self-discipline in Learning Online							
31	Sometimes, I skipped online teaching and learning unintentionally.	0.095	0.144	0.056	0.222	**0.8**	0.721
32	Sometimes, I played games or browse webpages online in studying time.	0.109	0.083	0.123	0.189	**0.77**	0.662
30	It is hard to focus on the course when learning online.	0.263	0.18	0.004	0.355	**0.625**	0.619

Target loadings are in boldface.

Table 4 presents the Cronbach’s alpha values as reliability index for each factor, and all the five factors have high reliability coefficients of 0.869 (factor 1), 0.888 (factor 2), 0.884 (factor 3), 0.833 (factor 4) and 0.797 (factor 5) respectively. Table 5 displays the descriptive statistics for the items loading on each demotivation factor. It can be discovered that the mean score of factor 4 (inappropriate learning environment) is the highest (2.84), followed by factor 3 (2.65), factor 1 (2.64) and factor 5 (2.54). The mean score of factor 2 (loss of task value) is the lowest (1.87).

**Table 4:** Cronbach alpha of each factor.

Factors	Cronbach’s α	N of items	*n*
F1	0.888	8	256
F2	0.869	8	256
F3	0.884	6	256
F4	0.833	4	256
F5	0.797	3	256

**Table 5:** Descriptive statistics for each factor.

Factor	No	*M*	*SD*	Skewness		Kurtosis	
				Value	*SE*	Value	*SE*
Factor 1: Low Expectancy for Success *(k = 8)*							
Total	256	2.64	0.59	−0.19	0.15	0.35	0.30
Factor 2: Loss of Task Value *(k = 8)*							
Total	256	1.87	0.53	0.01	0.15	−0.69	0.30
Factor 3: Negative Teacher Behavior *(k = 6)*							
Total	256	2.65	0.67	−0.47	0.15	0.59	0.30
Factor 4: Inappropriate Learning Environment *(k = 4)*							
Total	256	2.84	0.63	−0.16	0.15	0.23	0.30
Factor 5: Lack of Self-discipline in Learning Online *(k = 3)*							
Total	256	2.54	0.66	−0.02	0.15	0.21	0.30

### Differences in demotivating factors for English learning motivation, gender, and family background in blended learning context

**Figure 1:**
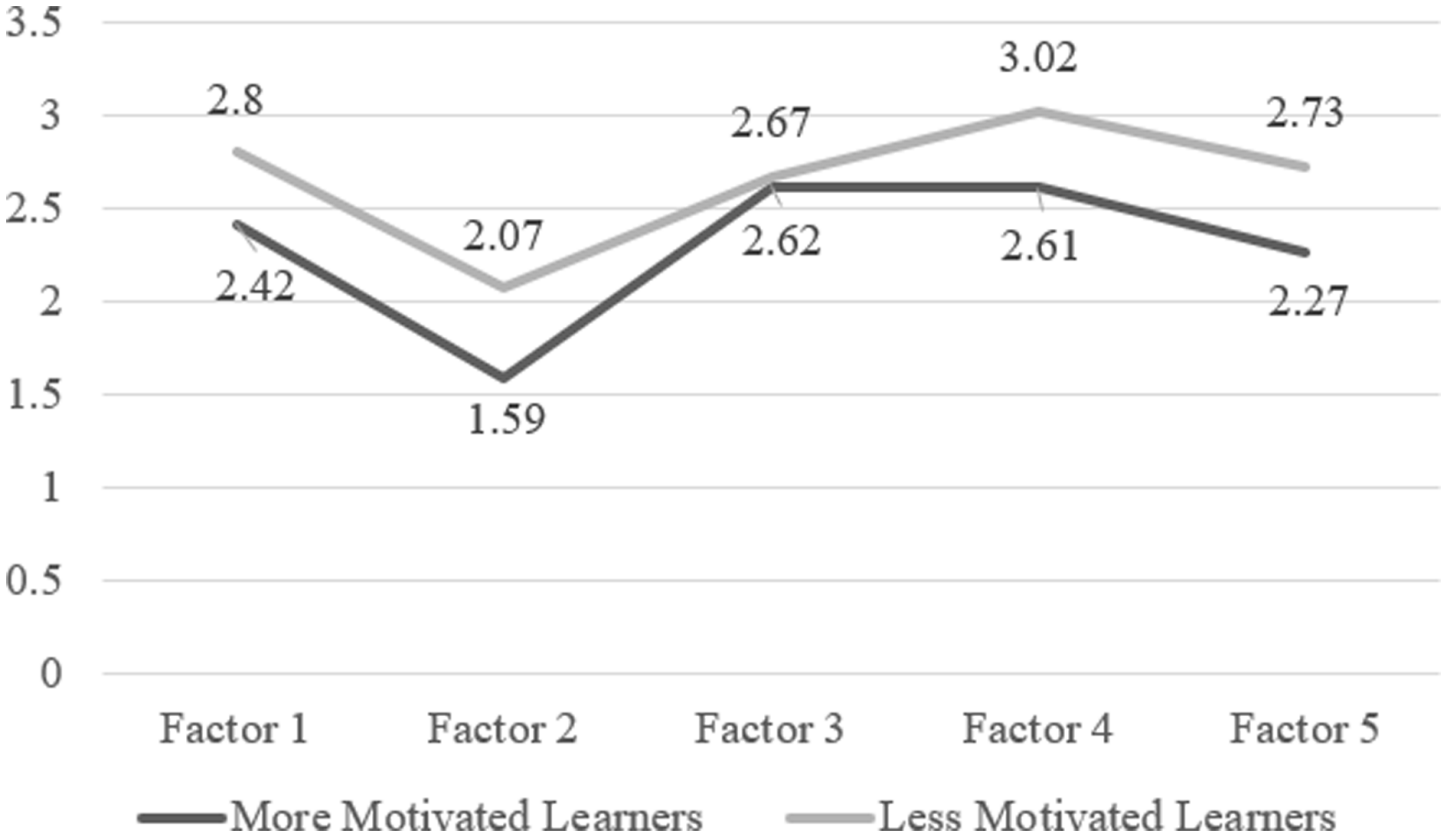
Mean scores of the five demotivating factors of more and less motivated learners.

Independent samples t-tests were implemented to examine whether differences exist between less motivated learners and more motivated over all the five factors and each factor. The results (see Table 6) show that there were statistically significant differences between the two groups for factor 1 (*t* = −5.439, *p* < 0.01), factor 2 (*t* = −7.954, *p* < 0.01), factor 4 (*t* = −5.418, *p* < 0.01), and factor 5 (*t* = −5.854, *p* < 0.01), whereas no statistically significant difference was found between the two groups for factor 3 (*t* = −0.527, *p* = 0.599). In other words, with reference to Figure 1 (mean scores of the five demotivating factors of more and less motivated learners), less motivated learners considered factor 1(Low Expectancy for Success), factor 2 (Loss of Task Value), factor 4 (Inappropriate Learning Environment), and factor 5 (Lack of Self-discipline in Learning Online) to be more demotivating than more demotivated learners, whereas no group difference was discovered for factor 3 (Negative Teacher Behavior).

**Table 6:** Independent samples t-test over learning motivation.

Factor	Learning motivation (Mean ± Std. D)		*t*	*p*
	more motivated(*n* = 108)	less motivated(*n* = 148)		
factor 1	2.418 ± 0.612	2.802 ± 0.517	−5.439	0.000
factor 2	1.589 ± 0.445	2.071 ± 0.502	−7.954	0.000
factor 3	2.622 ± 0.728	2.667 ± 0.627	−0.527	0.599
factor 4	2.606 ± 0.647	3.017 ± 0.561	−5.418	0.000
factor 5	2.275 ± 0.666	2.734 ± 0.584	−5.854	0.000

Similarly, Independent samples t-tests were adopted to examine whether there are differences between learners of different genders (male and female) and between learners from different family backgrounds (urban and rural) over each of the five factors. The results (see Table 7) show that no statistically significant difference was found between the male and female learners for all factors: factor 1 (*t* = 0.205, *p* = 0.838), factor 2 (*t* = −1.859, *p* = 0.064), factor 3 (*t* = 1.787, *p* = 0.075), factor 4 (*t* = −0.504, *p* = 0.615), and factor 5 (*t* = − 1.055, *p* = 0.292). Urban and rural learners did not show statistically significant difference for all factors (see Table 8): factor 1 (*t* = −2.437, *p* = 0.015), factor 2 (*t* = − 0.367, *p* = 0.714), factor 3 (*t* = − 0.289, *p* = 0.773), factor 4 (*t* = 0.706, *p* = 0.481), and factor 5 (*t* = −1.741, *p* = 0.083).

**Table 7:** Independent samples t-test over genders.

Factor	Gender (Mean ± Std. D)		*t*	*p*
	female(*n* = 152)	male(*n* = 104)		
factor 1	2.646 ± 0.560	2.631 ± 0.631	0.205	0.838
factor 2	1.817 ± 0.512	1.942 ± 0.559	−1.859	0.064
factor 3	2.709 ± 0.634	2.558 ± 0.713	1.787	0.075
factor 4	2.827 ± 0.565	2.868 ± 0.718	−0.504	0.615
factor 5	2.504 ± 0.608	2.593 ± 0.728	−1.055	0.292

**Table 8:** Independent samples t-test over family backgrounds.

Factor	Family background (Mean ± Std. D)		*t*	*p*
	urban(*n* = 116)	rural(*n* = 139)		
factor 1	2.542 ± 0.621	2.721 ± 0.552	−2.437	0.015
factor 2	1.851 ± 0.568	1.876 ± 0.503	−0.367	0.714
factor 3	2.635 ± 0.649	2.659 ± 0.692	−0.289	0.773
factor 4	2.871 ± 0.647	2.815 ± 0.616	0.706	0.481
factor 5	2.463 ± 0.662	2.607 ± 0.655	−1.741	0.083

## Discussion

The first research question asked what the demotivating factors for Chinese EFL college students in blended learning environment were. The factors extracted from the research were: (a) Low Expectancy for Success, (b) Loss of Task Value, (c) Negative Teacher Behavior, (d) Inappropriate Learning Environment, and (e) Lack of Self-discipline in Learning Online. We constructed our questionnaire on the basis of four factors (negative teacher behavior, loss of task value, low expectancy for success, and problems with learning environment), but one more factor was separated from them. Items concerning learners’ lack of self-discipline in learning online and inappropriate learning environment were hypothesized as one factor: problems with learning environment, but loaded as two independent factors. Other factors were retained the same as what they were presumed to be, with 5 items being deleted due to poor loading.

As it can be seen in Table 5, the mean score of Inappropriate Learning Environment factor is the highest (2.84), mean scores of Negative Teacher Behavior factor (2.65), Low Expectancy for Success factor (2.64), and Lack of Self-discipline in Learning Online factor (2.54) are between 2.5 to 2.7. In other words, these four factors are perceived as strong demotivating factors by Chinese EFL college students in blended learning environment. This finding lend support to many previous studies which showed factors related to learning environment (Gorham and Christophel, 1992; Christophel and Gorham, 1995; Gorham and Millette, 1997; Zhang, 2007; Sakai and Kikuchi, 2009; Kikuchi, 2011; Krishnan and Pathan, 2013; Xie et al., 2018; Adara et al., 2021; Xie et al., 2021; Huwari et al., 2023), teachers (Gorham and Christophel, 1992; Christophel and Gorham, 1995; Gorham and Millette, 1997; Zhang, 2007; Katt and Condly, 2009; Kikuchi and Sakai, 2009; Krishnan and Pathan, 2013; Xie et al., 2018; Adara et al., 2021; Xie et al., 2021; Huwari et al., 2023), intrinsic motivation (Gorham and Christophel, 1992; Christophel and Gorham, 1995; Gorham and Millette, 1997; Zhang, 2007; Sakai and Kikuchi, 2009; Kikuchi, 2011; Krishnan and Pathan, 2013; Çankaya, 2018; Xie et al., 2018, 2021) are strong demotivating factors. However, the mean score of Loss of Task Value factor is the lowest (1.87). To be specific, 82.2% of the participants chose 1 or 2 for items constituting this factor, which means those participants did not perceive this factor as demotivating. Thus, although this factor (factor 2) was extracted as a demotivating factor, the majority of the participants (82.2%) did not consider it to be demotivating. This factor was borrowed from Xie’s research (Xie et al., 2018, 2021), however, results from this paper show that most participants did not consider it as demotivating. In other words, most participants believe English and learning English are of great value, which could be supported by the fact that more than 1.35 billion people around the world speak English and about 360 million people speak English as their first language, and English is the most commonly spoken and studied foreign language in the world (Lyons, 2021).

The second research question asked about whether these demotivating factors differ for English learning motivation (less motivated and more motivated), gender (male and female), and family background (rural and urban). The results showed there were no statistically significant difference between male and female learners, and between rural and urban learners for each of the five factors. In other words, male and female participants perceived these five demotivating factors similarly demotivating, so did rural and urban participants. This finding echoed some previous studies (Çankaya, 2018; Santosa and Riady, 2021) which believed that gender does not influence demotivation factors.

Whereas statistically significant differences were detected between less motivated and more motivated learners for factor 1(Low Expectancy for Success), factor 2 (Loss of Task Value), factor 4 (Inappropriate Learning Environment), and factor 5 (Lack of Self-discipline in Learning Online). That means participants with almost no motivation and with a little motivation considered these four factors to be more demotivating than participants with moderate motivation and with high motivation. No statistically significant difference between less motivated and more motivated learners was discovered for factor 3 (Negative Teacher Behavior), which means participants with almost no motivation and with a little moderate, participants with moderate motivation and with high motivation found negative teacher behaviors as equally demotivating. This finding is well in accordance with Sakai & Kikuchi’s study (Sakai and Kikuchi, 2009), except the newly discovered factor Lack of Self-discipline in Learning Online.

### Limitations and directions for future research

Although the authors believe this paper contributes significantly to research on EFL learners’ demotivation in blended learning environment, several limitations exist. The First limitation would be the sample size and variety, which should be enlarged and enriched in future investigations. Second, even though some variables which may exert influence to demotivating factors in the study were analyzed, more variables could be taken into consideration. Also, there is a lack of qualitative analysis to augment the results of this research. Finally, there is a shortage of studies covering this research theme, EFL learners’ demotivation in blended learning environment, which may cause this present research not thorough or solid enough.

## Conclusion

In the present research, EFL learners’ demotivating factors in blended learning context were investigated. One novel factor, learners’ lack of self-discipline in learning online, was detected among the five extracted factors. It was also discovered that gender and family background do not influence demotivation factors, whereas less motivated learners tend to perceive most demotivating factors as stronger. Based on the findings of the study, to reduce the occurrence of EFL learners’ demotivation in blended learning environment, EFL learners and educators are suggested to cope with the problem from the perspective of raising EFL learners’ motivation level and resisting temptations from the internet.

## Data availability statement

The raw data supporting the conclusions of this article will be made available by the authors, without undue reservation.

## Author contributions

WZ: Conceptualization, Methodology, Software, Writing – original draft. YZ: Investigation, Resources, Writing – review & editing. EJ: Conceptualization, Project administration, Supervision, Writing – review & editing.
