# Supplementary material for: Analyzing EFL learners’ demotivating factors in blended learning context
Source: Front Psychol. 2023 Oct 20;14:1290034. doi: 10.3389/fpsyg.2023.1290034 (PMC10623303; doi:10.3389/fpsyg.2023.1290034)
Supplement: Supplementary file 1 [file Data_Sheet_1.pdf]

Appendix A

Learner Perceptions of Demotivator Scale in Blended Learning Environment

This is for the research to investigate English learning in a blended context. Your personal information will be protected. Please share your honest feeling towards the questions bellow. Thank you for your cooperation.					
1	gender:	(1).Male	(2).Female		
2	major:	( )			
3	proportion of content delivered and learned online:	(1).0-29%	(2).30%-50%	(3).51%-79%	(4).80%-100%
4	CET4 score:	( )			
5	family background :	(1). City	(2). Countryside		
6	How motivated are you to learn English in a blended learning context?	(1). I have almost no motivation	(2). I have a little motivation	(3). I have moderate motivation	(4). I have high motivation
7	Have you ever experienced times unwilling to learn English in a blended learning context? When and where if so.	(1).Yes, when ____________ ________________________ ________________________		(2).Never	

Please indicate your degree of agreement to the following sentences.

		Strongly Disagree	Disagree	Agree	Strongly Agree
8	~~The learning objectives are not clear for the class.~~	1	2	3	4
9	The only purpose of learning English is to pass all the exams.	1	2	3	4
10	I don’t see the value of learning English.	1	2	3	4
11	Learning English takes forever, and it may not get you anywhere.	1	2	3	4
12	I wonder why English is needed in a monolingual country.	1	2	3	4
13	English has no use for my major.	1	2	3	4
14	I’m not interested in English at all.	1	2	3	4
15	It’s not clear to me why I must learn English.	1	2	3	4
16	I take English class only because it’s a required class.	1	2	3	4
17	~~If learning English means losing a fun life, I’ll choose the latter one.~~	1	2	3	4
18	I seriously don’t know how to speak English fluently.	1	2	3	4
19	I have made many attempts to learn English, but I have not improved.	1	2	3	4
20	I’m not aware of the strategies to improve my listening skills.	1	2	3	4
21	I really want to master English, but I don’t know how.	1	2	3	4
22	I haven’t found an effective way to learn English.	1	2	3	4

23	English grammar is tough and confusing.	1	2	3	4
24	Reading comprehension articles are hard to understand.	1	2	3	4
25	I struggle with improving my English writing skills.	1	2	3	4
26	I do not deal with real language situations under blended teaching and learning environment.	1	2	3	4
27	I seldom have opportunities to practice English.	1	2	3	4
28	I do not collaborate with classmates in learning English.	1	2	3	4
29	I do not communicate with classmates about English learning.	1	2	3	4
30	It is hard to focus on the course when learning online.	1	2	3	4
31	Sometimes, I skipped online teaching and learning unintentionally.	1	2	3	4
32	Sometimes, I played games or browse webpages online in studying time.	1	2	3	4
33	~~I do not like learning offline combining online approach.~~	1	2	3	4
34	~~Online learning caused my eyes fatigue and eyesight loss.~~	1	2	3	4
35	~~It is hard for me master the technology of learning online.~~	1	2	3	4

Please indicate your demotivating level to the following scenarios.

		Not Demotivating at all	Not Demotivating	Demotivating	Very Demotivating
36	Teachers are not responsive to our learning needs.	1	2	3	4
37	Teachers don't have a sense of responsibility for the teaching job.	1	2	3	4
38	Teachers don't have faith in their students.	1	2	3	4
39	Teachers are not inspiring or encouraging.	1	2	3	4
40	Teachers reward performance rather than learning.	1	2	3	4
41	Teachers seldom motivate us to learn.	1	2	3	4
