# Supplementary figures and images for: Analyzing EFL learners’ demotivating factors in blended learning context
Source: Front Psychol. 2023 Oct 20;14:1290034. doi: 10.3389/fpsyg.2023.1290034 (PMC10623303; doi:10.3389/fpsyg.2023.1290034)

## Appendix B: scree plot

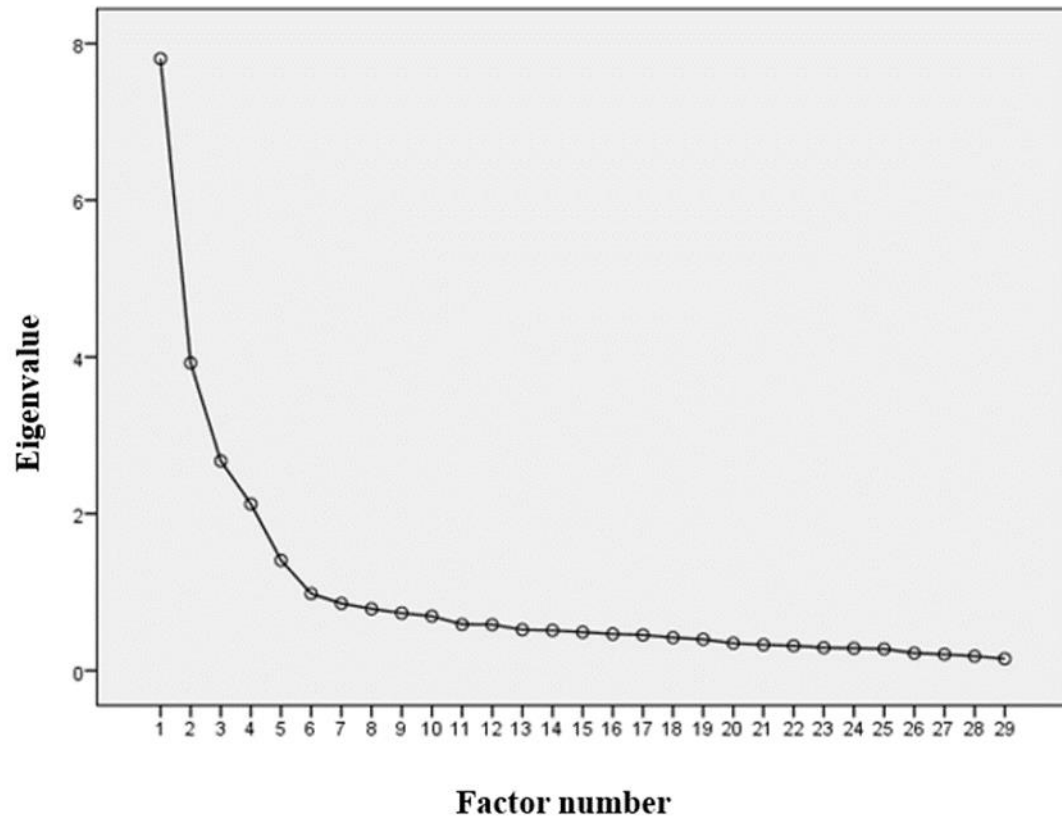

Supplement: Supplementary file 2 [file Image_1.pdf]
